# Reopening Motor Learning Windows: Targeted Re-Engagement of Latent Pathways via Non-Invasive Neuromodulation

**DOI:** 10.3390/life16030506

**Published:** 2026-03-19

**Authors:** Diego Mac-Auliffe, Akhil Surapaneni, José del R. Millán

**Affiliations:** 1 Center for Perceptual Systems, Department of Psychology, The University of Texas at Austin, Austin, TX 78712, USA; 2 Chandra Family Department of Electrical and Computer Engineering, Cockrell School of Engineering, The University of Texas at Austin, Austin, TX 78712, USA; akhilsurapaneni@utexas.edu (A.S.); jose.millan@austin.utexas.edu (J.d.R.M.); 3 Department of Neurology, Dell Medical School, The University of Texas at Austin, Austin, TX 78712, USA

**Keywords:** Hebbian plasticity, spike-timing-dependent plasticity, motor learning, neuromodulation, brain–computer interface, closed-loop stimulation, cortical–spinal connectivity, neurorehabilitation, motor recovery, plasticity windows

## Abstract

Motor recovery after stroke, spinal cord injury, or traumatic brain injury reflects relearning rather than simple restitution, as surviving circuits retain plastic potential that can be re-engaged through temporally precise stimulation. This review synthesizes convergent findings demonstrating that Hebbian and spike-timing-dependent mechanisms govern reorganization across cortical, striatal, and spinal levels. Leveraging these timing rules to shape excitability during receptive network states enables durable changes in connectivity and behavior. This effect depends on temporal precision, physiological state, and reinforcement—not stimulus intensity alone—within plasticity windows regulated by metaplastic mechanisms that determine whether Hebbian processes are expressed. Together, these principles define a translational framework for neurorehabilitation, emphasizing biomarker-guided, adaptive, and scalable strategies aligned with intrinsic rules of experience-dependent reorganization.

## 1. Introduction

Motor recovery after stroke, spinal cord injury (SCI), or traumatic brain injury (TBI) is not simply about regaining lost function. It reflects relearning—a reassembly of movement supported by the nervous system’s capacity to adapt and reorganize. Evidence from animal and human studies suggests that recovery often involves a reweighting and unmasking of pre-existing neural architectures rather than the emergence of entirely new cortical computations. Previously underutilized pathways and surviving networks may thus contribute to functional compensation [1,2].

In recent years, non-invasive neuromodulation has emerged as a promising approach to guide this process. By modulating cortical excitability and aligning stimulation with ongoing neural activity, several techniques seek to induce transient “plasticity windows,” resembling periods of heightened sensitivity during early development and following brain injury. When paired with voluntary movement or sensorimotor feedback, these interventions may extend beyond short-term excitability shifts, potentially promoting more durable changes in network communication through timing-dependent mechanisms consistent with Hebbian and spike-timing-dependent plasticity (STDP).

Recovery therefore depends less on isolated regions than on dynamic interactions across the motor network. Focal lesions produce distributed effects, disrupting connected but distant areas. Structural and functional neuroimaging studies support this systems-level view: even small lesions can fragment the connectome and impair long-range coordination [3,4]. Graph-theoretical analyses indicate that such injuries reduce “small-world” efficiency—the balance between local clustering and global integration—and that restoration of connectivity is associated with functional recovery [5,6]. When major descending pathways such as the corticospinal tract (CST) are compromised, polysynaptic alternatives, including cortico-reticular and cortico-cerebellar routes, may remodel and assume partial control [7,8]. Activity within the unaffected hemisphere, particularly in parietal and premotor regions, may also contribute to movement re-coordination [9].

Experimental evidence suggests that neuromodulation can support this relearning process by biasing excitability during behaviorally relevant moments—such as movement onset, motor imagery, or favorable oscillatory phases—thereby transforming stimulation into a Hebbian reinforcement signal [10,11]. Longitudinal observations further indicate that, in the absence of intervention, network organization may drift toward increased segregation [6].

Here, we propose that motor recovery may be conceptualized as a form of relearning that can be facilitated when neuromodulation is delivered in a temporally precise and state-dependent manner. Aligning stimulation with endogenous excitability dynamics and behavioral intent may help recruit previously underactive pathways and strengthen task-relevant circuits—not through indiscriminate excitation, but through reinforcement mechanisms grounded in established principles of synaptic plasticity. This review integrates mechanistic evidence across cortical, striatal, and spinal levels to examine how neuromodulation may, under appropriate conditions, support longer-term network reorganization. By tracing these processes, we outline a framework linking experimental physiology to translational rehabilitation, consistent with the view that therapeutic neuromodulation engages timing- and state-sensitive mechanisms that also govern motor learning in the intact nervous system. Key conceptual terms are summarized in a glossary for clarity.

## 2. Mechanistic Foundations of Hebbian Plasticity in the Motor Pathway

Motor learning and rehabilitation rely on experience-dependent plasticity, whereby synaptic connections are strengthened or weakened through coincident pre- and postsynaptic activity [12]. In humans, this principle is exemplified by paired associative stimulation (PAS), which combines peripheral nerve input with transcranial magnetic stimulation (TMS) over the motor cortex (M1) at precisely timed intervals to induce timing-dependent plasticity. When a TMS pulse reaches M1 roughly 25 ms after median-nerve stimulation, motor-evoked potentials (MEPs) increase, consistent with long-term-potentiation-like (LTP) facilitation [13]. Reversing the order yields long-term-depression-like (LTD) suppression, reflecting the spike-timing-dependent plasticity (STDP) rule first described in animal models [14,15]. These effects are topographically specific to the conditioned muscle and confined to a 25–35 ms window, highlighting the millisecond-scale temporal sensitivity of human synapses [16].

These Hebbian mechanisms extend beyond cortex to the spinal interface. Paired corticomotoneuronal stimulation (PCMS) synchronizes a TMS-evoked descending volley with a peripheral-nerve-evoked antidromic spike, allowing both signals to reach spinal α-motoneurons within ±2 ms. When cortical activity precedes motoneuron depolarization by ∼1 ms, corticospinal transmission strengthens; reversing the order produces weakening [17]. This bidirectional STDP-like window depends on NMDA-receptor-mediated calcium entry at the motoneuron, indicating that spinal synapses follow timing-sensitive coincidence rules similar to those observed in cortex [18]. Functionally, PCMS can enhance motor output for approximately 90 min and improve finger-abduction strength even in chronic SCI [19,20]. Pairing PCMS with slight voluntary contraction further boosts motoneuron depolarization, suggesting that volitional drive may gate spinal plasticity.

At the local-circuit level, inhibitory tone influences the expression of plasticity. Short-interval intracortical inhibition (SICI), a measure of inhibitory control over motor cortex output, can be assessed using paired-pulse TMS: a weak conditioning pulse delivered 2–5 ms before a stronger test pulse suppresses the resulting MEP. The resulting SICI reflects GABA_*A*_-mediated intracortical inhibition within M1; individuals with stronger baseline SICI tend to show reduced MEP amplitudes and attenuated LTP-like responses to PAS, whereas lower inhibition is associated with greater facilitation [21,22]. Voluntary movement or brief practice can transiently reduce SICI, shifting the circuit toward a state more permissive for subsequent potentiation [23].

The Bienenstock–Cooper–Munro (BCM) model [24] offers a theoretical framework for plasticity regulation through a sliding modification threshold, denoted $\theta_{m}$. In this model, prior neural activity sets the level of $\theta_{m}$: low activity lowers the threshold and favors LTP, whereas sustained high activity raises it and biases synapses toward LTD. This adjustment stabilizes average firing rates while preserving sensitivity to subsequent input. Evidence from animal and computational studies suggests that such threshold shifts may involve changes in NMDA-receptor subunit composition [25] and modulation of voltage-gated ion channels [26]. This activity-dependent modulation of future capacity for synaptic change is commonly referred to as metaplasticity.

Evidence for metaplasticity in humans extends beyond timing-based protocols. Repeated anodal tDCS delivered in close succession can invert its after-effects, consistent with a transient elevation of $\theta_{m}$ that constrains subsequent plasticity [27]. When stimulation is spaced over longer intervals, plastic capacity appears to recover, consistent with threshold recalibration. Rosenkranz et al. [28] showed that one day of thumb-movement practice elevated corticospinal excitability and reduced SICI, yet PAS produced LTD-like suppression. After five days, PAS responsiveness normalized and motor-map organization refined, consistent with a transient upward shift of $\theta_{m}$ during early practice that recalibrated as consolidation progressed. These findings imply that intense activity may transiently narrow the available LTP range, with recovery emerging as reorganization stabilizes.

At the cellular level, brain-derived neurotrophic factor (BDNF) acts as a key modulator of this balance. BDNF enhances NMDA-dependent Ca^2+^ entry and reduces inhibitory tone, effects associated with lowering $\theta_{m}$ and facilitating LTP induction and consolidation. Reduced BDNF release is associated with elevated $\theta_{m}$ and a bias toward LTD or short-lived plastic changes. A common BDNF polymorphism (Val66Met), which limits activity-dependent secretion, has been linked to weaker and less durable LTP-like after-effects [29]. Because BDNF interacts with both NMDA signaling and GABAergic inhibition, such genetic variation may help explain why some individuals appear “non-responsive.” In most cases, plasticity mechanisms remain intact but are functionally gated by inhibition, recent activation, or neuromodulatory state.

These observations suggest that functional limitation after injury may reflect not the absence of Hebbian mechanisms, but the state in which they are expressed. Neuromodulation may shift the modification threshold ($\theta_{m}$) toward a range that allows practice and feedback to drive more stable synaptic and behavioral change. From this perspective, metaplasticity influences whether stimulation produces durable effects; when the system is not in a receptive state, Hebbian timing rules may fail to translate into functional recovery.

Plasticity at the synaptic and circuit level ultimately manifests in observable behavior. Repetitive activation—through use or stimulation—can enlarge the cortical representation of trained muscles, whereas disuse leads to contraction of these maps [30,31]. In animal models, skilled-reach training induces LTP-like synaptogenesis and dendritic-spine remodeling [32]. Comparable effects have been observed in humans, where facilitatory PAS expands the excitability map of the conditioned muscle [33]. These cortical changes occur within broader networks, interacting with cerebellar and basal ganglia circuits that refine timing, error correction, and consolidation [34].

With continued practice, control may gradually shift from cortical to subcortical structures—a transition demonstrated in rodents, where M1 lesions impaired learning but spared execution once skills were established [35]. In this framework, the motor cortex acts as a tutor: it guides subcortical and spinal circuits through repeated, temporally coordinated activation, enabling lower networks to sustain the learned sequence more independently. Such hierarchical redistribution has been proposed to support both skill automatization and aspects of post-injury recovery. Following injury, surviving networks may retain latent pathways that can be re-engaged through task-specific, timing-contingent stimulation. Protocols such as PAS [13,33] and PCMS [19,36] may enhance training-related gains by reinforcing appropriate corticospinal co-activation during movement.

## 3. State- and Phase-Dependent Neuromodulation

Phase- and state-dependent stimulation applies these gating principles by aligning external input with endogenous excitability dynamics. In the motor system, β- and μ-band rhythms organize such fluctuations: TMS pulses delivered at the depolarizing phase evoke larger MEPs [37]. EEG-triggered studies further show that pulses timed to the negative peak of the μ cycle—when inward current depolarizes the membrane—maximize corticospinal output, whereas stimulation at the opposite phase produces weaker responses [38,39]. Delivering stimulation during depolarizing phases may bias otherwise variable inputs toward reproducible modulation [40,41], although cortical evoked responses and corticospinal output do not always exhibit identical sensitivity to oscillatory phase or power [42].

Because conduction and hardware delays can shift the effective stimulation phase, phase-locked protocols require prediction of the upcoming oscillatory state rather than simple reaction to the last measured cycle. Predictive approaches use autoregressive modeling to extrapolate ongoing EEG or MEG rhythms and estimate the upcoming oscillatory phase with sub-cycle precision [41,43]. High-fidelity recording with low-impedance electrodes and careful spatial referencing minimizes phase error [38]. Subject-specific calibration can further compensate for traveling-wave distortions. Recent educated temporal prediction approaches incorporate individual dominant frequencies and conduction delays, delivering stimulation only when oscillatory power permits reliable phase estimation [43,44]. A similar anticipatory logic guides adaptive spinal protocols in which stimulation is synchronized to gait or grip phase, maximizing coincidence between descending commands and afferent feedback [45,46]. Such predictive control aims to restore millisecond-scale precision in pairing stimulation with endogenous excitability, thereby reinforcing intrinsic temporal coding mechanisms.

γ-band tACS applied simultaneously over M1 and contralateral cerebellum, with phase offsets adjusted to account for conduction delays, produces maximal visuomotor gains when the regions are stimulated in anti-phase [47]. Closed-loop brain–computer interface (BCI) systems paired with functional electrical stimulation (FES)—which activates peripheral nerves or muscles to generate movement—translate this principle to the behavioral level. Pairing motor-intention-related μ-desynchronization with time-locked neuromuscular stimulation has been associated with strengthened effective connectivity within the lesioned hemisphere and improved motor performance in stroke patients [48]. When cortical intent is detected, the resulting proprioceptive feedback may reinforce the same neural pattern.

Although precise timing determines when stimulation can act, its sustained impact depends on maintaining excitability within a responsive range. Adaptive controllers that measure ongoing brain state and adjust stimulation parameters in real time may approximate endogenous regulatory processes, helping preserve responsiveness without destabilizing network activity [41,49,50,51]. When excitability is stabilized, stimulation may interact more effectively with ongoing sensorimotor dynamics across cortical and peripheral circuits [3,41,43]. Taken together, these findings support the presence of reliable phase- and state-dependent modulation of corticospinal excitability.

## 4. Network Re-Engagement and Interhemispheric Rebalancing

Beyond synaptic and hierarchical redistribution, recovery also unfolds at the level of large-scale interhemispheric dynamics. In healthy individuals, unimanual movement relies on inhibitory coupling from the active to the resting M1 to prevent mirror activity. Premotor and supplementary motor area (SMA) nodes coordinate facilitation within the engaged hemisphere [52]. After stroke, this balance is altered: excitatory drive from premotor and SMA areas to the lesioned M1 is reduced, while the contralesional M1 may exert increased transcallosal inhibition that suppresses residual output [53].

Early neuroimaging studies showed that movements of the paretic limb recruit bilateral premotor and parietal regions. When contralesional activity predominates, this compensatory pattern has been associated with poorer functional outcomes [54,55]. Even small M1 lesions can propagate through the motor network, altering connectivity among premotor, supplementary, parietal, cerebellar, and basal ganglia regions that normally cooperate during voluntary control [56]. In cases of spontaneous recovery, activity and connectivity within perilesional M1, premotor cortex, and SMA tend to increase, while contralesional hyperactivity declines [55,56]. However, restoration of basic motor capacity remains variable and depends in part on the extent of both superficial and deep structural damage.

Rehabilitation and sustained practice may gradually rebalance this pattern, as intrahemispheric facilitation strengthens and transcallosal inhibition diminishes [4,57]. This adaptive reorganization is often accompanied by progressive re-lateralization of activity toward the affected hemisphere [58]. The SMA appears to function as a context-dependent hub, modulating cooperation and suppression according to task demands [59,60]. These changes have been associated with reinforcement of spared tracts and improved behavioral performance [3,41,47]. Consistent with this framework, non-human primate studies suggest that recovery after cortical injury involves a re-separation of β-dominated resting activity from neural patterns that effectively drive movement [61].

Non-invasive stimulation can shift these network dynamics in a similar direction. High-frequency rTMS or anodal tDCS applied over the affected M1 can increase cortical excitability and descending output, whereas low-frequency or cathodal stimulation of the contralesional hemisphere may reduce excessive interhemispheric inhibition [62,63,64,65]. When combined with task-specific training, such approaches may help stabilize transient excitability changes and promote more sustained re-lateralization of motor control.

BCI systems and other contingent neuromodulation approaches that couple motor-related desynchronization to orthosis movement or proprioceptive feedback have likewise been associated with cortical re-lateralization and clinical improvement [48,66,67,68,69,70,71]. Comparable associative principles extend to spinal stimulation paradigms [46,72,73]. Such pairing has been linked to more consistent corticospinal output and coordinated network activity [46,74,75].

Strengthened intrahemispheric β/γ coupling, restored inter-M1 β-phase coupling, and rebalanced transcallosal dynamics correlate with functional improvement [55,56,76,77]. Functional MRI studies similarly report a shift of activity toward perilesional regions [67,68,78]. At the network level, topology appears to evolve from diffuse and inefficient configurations toward small-world organization, characterized by more balanced integration and segregation [79]. Together, these changes reflect restoration of coordinated motor network dynamics, potentially creating conditions favorable for stabilizing learned patterns.

Nevertheless, these recovery trajectories are not uniform. An early post-injury phase in humans is often characterized by heightened cortical excitability, paralleled in animal models by structural and synaptic remodeling within peri-infarct cortex [80]. In such models, reduced inhibition and enhanced NMDA-dependent signaling accompany broader synaptic reorganization, reflected in increased network responsiveness [81]. Even during chronic stages, state-contingent approaches such as BCI or spinal stimulation promote re-engagement of previously underactive pathways [46,48,73].

These findings indicate that large-scale network rebalancing remains constrained by underlying corticospinal integrity and excitability. Biomarker-guided frameworks, such as the Predicting REcovery Potential (PREP) algorithm [82], formalize the role of CST integrity and excitability in shaping recovery potential and provide a structured basis for patient stratification. In a randomized pilot study of subacute stroke patients with severe upper-limb paresis, Brunner et al. [83] reported that baseline CST integrity, indexed by MEP presence, was the strongest predictor of clinically meaningful improvement. Together, these results emphasize the importance of stratifying severely impaired cohorts when evaluating BCI–FES interventions. However, restoration of interhemispheric balance and cortical re-lateralization does not by itself ensure durable motor learning. Stabilization of repeated activity patterns into consistent behavior requires reinforcement-dependent mechanisms mediated by cortico-striatal circuits.

## 5. Cortico-Striatal Mechanisms of Human Motor Learning

Restored cortical dynamics provide the foundation for re-engagement, yet consolidation into learned behavior depends on cortico-striatal circuits that stabilize repeated cortical activity into consistent motor output. Motor learning progresses through a redistribution of control across the motor hierarchy, beginning with flexible cortical engagement and advancing toward cortico-striatal automation. Early in practice, distributed frontoparietal and associative networks—including prefrontal, premotor, and SMA regions—support sequencing, attention, and error correction [84,85,86,87,88,89]. Concurrent activity in the anterior striatum and rostral putamen reflects this cognitive load [34]. With continued training, activation shifts toward posterior sensorimotor territories, marking the emergence of more automatized control [34,88]. Lesion and electrophysiological studies in non-human primates support this gradient: disruption of anterior striatum impairs acquisition but spares execution, whereas posterior lesions abolish established sequences [90]. Repeated, temporally coordinated co-activation between cortical and striatal neurons strengthens synaptic efficacy through STDP, forming stable sensorimotor representations that support efficient execution of learned actions [87,91].

Within the cortex, early practice is accompanied by transient increases in excitability and reductions in inhibition. After a single session, M1 excitability rises and GABA_*A*_-mediated inhibition decreases, yet externally applied PAS can fail to induce additional potentiation, consistent with temporary saturation of plastic capacity [28]. Magnetic resonance spectroscopy studies further show that the degree of disinhibition correlates with learning rate [23]. Across days of practice, cortical map expansion in humans and synaptogenesis in animal models accompany the stabilization of motor skills [32,92]. Together, these observations suggest that early performance gains draw on existing synaptic resources, whereas long-term retention depends on structural remodeling. This cortical adaptation unfolds within distributed cortico-subcortical loops that influence whether transient improvements consolidate into durable motor skills.

Cerebellar–striatal interactions contribute to this learning architecture. The cerebellum supports early, error-based calibration, engaging lobules V–VI and dentate nuclei to refine timing and accuracy [88,93], whereas the basal ganglia integrate reinforcement signals that stabilize successful action patterns [87,94]. Lesion studies support this functional distinction: cerebellar damage disrupts adaptation to novel dynamics or visuomotor perturbations, while striatal injury impairs habit formation. As performance stabilizes, control migrates from cerebellar-dominated error processing toward sensorimotor territories of the putamen, reflecting the transition from flexible correction to automated execution [34,88]. Cerebellar tDCS has been shown to facilitate this transition, potentially by modulating cerebellar output and cortico-striatal communication [95]. Motivational context further influences the sequence: punishment enhances short-term cerebellar error sensitivity, whereas reward promotes dopaminergic plasticity in M1 and striatum, supporting longer-term retention [96].

Dopamine acts as a key reinforcement-related modulator linking these learning phases. Phasic midbrain release, particularly from the ventral tegmental area (VTA), facilitates LTP in both M1 and striatum in animal models [97], and in humans transforms transient potentiation into more stable plastic change when movements are paired with reward [98]. VTA lesions impair skill acquisition, whereas local dopamine restoration reinstates learning [97]. At the synaptic level, D1-receptor activation enhances NMDA-dependent LTP and expands the dynamic range of modification [99]. Through intracellular signaling cascades, dopamine supports late-phase plasticity and stabilization of synaptic structure. Human studies further indicate that repetition paired with reward yields stronger and longer-lasting plasticity than repetition alone [98]. Pharmacological evidence suggests an inverted-U relationship between dopaminergic tone and facilitation: optimal levels prolong plasticity, whereas both deficient and excessive states diminish it [100,101,102,103]. Together, these findings position dopamine as a regulator of whether coincident activity stabilizes into long-term consolidation across cortico-basal-ganglia–thalamo-cortical circuits.

As learning consolidates, control shifts from cortical exploration to subcortical reinforcement circuits. Sensorimotor territories of the basal ganglia increasingly support execution, reducing the need for continuous cortical supervision. The motor cortex generates exploratory patterns and instructive signals that are refined through repeated cortico-striatal and cerebellar interaction until execution becomes more autonomous [104,105]. In rodents, motor cortex is required for acquisition but not for performance once sequences are established; lesions before training prevent learning, whereas lesions after training spare execution [35]. Human neuroimaging reflects a similar progression: early learning recruits widespread prefrontal, premotor, and associative striatal territories, which gradually narrow to SMA, posterior putamen, and ventrolateral thalamus as performance stabilizes [34,88,106]. These cortico-striatal dynamics ultimately depend on reliable descending transmission through corticospinal and propriospinal pathways. When this communication axis is compromised, stabilized cortical commands cannot be effectively conveyed to spinal circuits, limiting the translation of consolidated learning into functional motor output.

## 6. Re-Engaging Spinal Networks Through Targeted Neuromodulation

Motor recovery depends on restoring effective communication between cortical intent and the spinal circuits that execute movement. In a first-in-human study, Powell et al. [72] reported that lateral epidural stimulation of the cervical spinal cord (C4–T1) can amplify residual cortical commands to produce functional arm and hand movements years after stroke, primarily by increasing the excitability of spinal interneuronal and motoneuronal networks rather than directly activating muscles. Notably, some improvements persisted after stimulation, consistent with activity-dependent strengthening of spared corticospinal pathways rather than purely assistive effects. Building on this principle, Lorach et al. [107] linked cortical motor signals directly to spinal stimulation via an implanted brain–spine interface, showing that real-time coupling of intent and spinal activation can support more physiologically patterned movement. In this framework, spinal neuromodulation does not replace cortico-striatal learning processes, but restores the descending communication channel required for those processes to shape motor execution.

Non-invasive approaches can produce comparable effects by increasing segmental excitability through afferent recruitment. In a multicenter trial, Moritz et al. [108] demonstrated that transcutaneous cervical stimulation paired with task-specific rehabilitation led to sustained improvements in arm and hand function in individuals with chronic tetraplegia. Stimulation alone produced only transient facilitation, whereas coupling stimulation with voluntary effort was associated with more persistent gains. Similarly, Oh et al. [46] found that transcutaneous spinal stimulation combined with task-specific grip training enhanced hand motor output more effectively than stimulation or training alone, supporting the view that spinal neuromodulation is most effective when temporally aligned with motor intent.

This principle extends beyond central lesions. In peripheral nerve injury, targeted tSCS has been associated with engagement of dormant pathways. Chandrasekaran et al. [74] reported substantial recovery of thumb-flexion force and renewed tactile sensation months after therapy cessation, suggesting that coordinated spinal excitation and voluntary drive may promote durable sensorimotor reintegration even outside central nervous system injury.

Recent work suggests that stimulation need not evoke overt contractions to reorganize spinal network function. Taccola et al. [49] described this as electrical enabling motor control: modest depolarization increases network responsiveness without imposing output. Under these conditions, residual descending commands and proprioceptive feedback can once again shape coordinated activity through intrinsic spinal sensorimotor dynamics. Human studies parallel these mechanisms. Hastings et al. [109] and Gad et al. [110] showed that pairing sub-threshold stimulation with activity-based therapy improves posture and coordination without visible contractions, with benefits that persist across sessions. Sub-threshold protocols may also modulate supraspinal learning dynamics. Alawieh et al. [111] found that inhibitory cervical stimulation enhanced *µ*-band desynchronization and accelerated BCI skill acquisition in both healthy and spinal cord–injured participants. Complementing these findings, Xu et al. [112] review evidence that spinal direct-current stimulation elevates BDNF expression and shifts excitatory–inhibitory balance, potentially providing a biochemical substrate for reinforcement of sensorimotor loops. In this view, low-intensity stimulation may restore dynamic range for learning while preserving endogenous pattern generation.

Because spinal control is distributed along the neuraxis rather than confined to a single segment, recovery may benefit from synchronizing excitability across multiple spinal levels. Multi-site stimulation over cervical, thoracolumbar, and lumbosacral regions increases the excitability of interconnected networks linking trunk, proximal, and distal motor pools. These propriospinal circuits coordinate communication between segments, allowing postural stabilization to support distal stepping movements. Clinical studies in paraplegic and stroke populations report improved step symmetry, trunk control, and inter-limb coordination under such distributed paradigms, consistent with more integrated rostrocaudal network dynamics [73,113]. Mechanistically, concurrent depolarization of ascending propriospinal neurons and descending reticulospinal projections may enhance coupling between brainstem centers and spinal motor pools, supporting more coherent sensorimotor integration across the axis.

Durable recovery may be more likely when cortical and spinal excitability are tuned together. Pairing transcranial and transspinal stimulation temporally aligns activation across supraspinal and segmental levels, potentially strengthening corticospinal output while increasing spinal network responsiveness in a manner consistent with spike-timing principles. In rodents, Sharif et al. [114] and subsequent analyses by Xu et al. [112] indicate that combining intermittent theta-burst TMS with transspinal direct-current stimulation synchronized excitability shifts across the motor hierarchy and was associated with greater axonal sprouting and faster behavioral recovery than either intervention alone.

**Figure 1:**
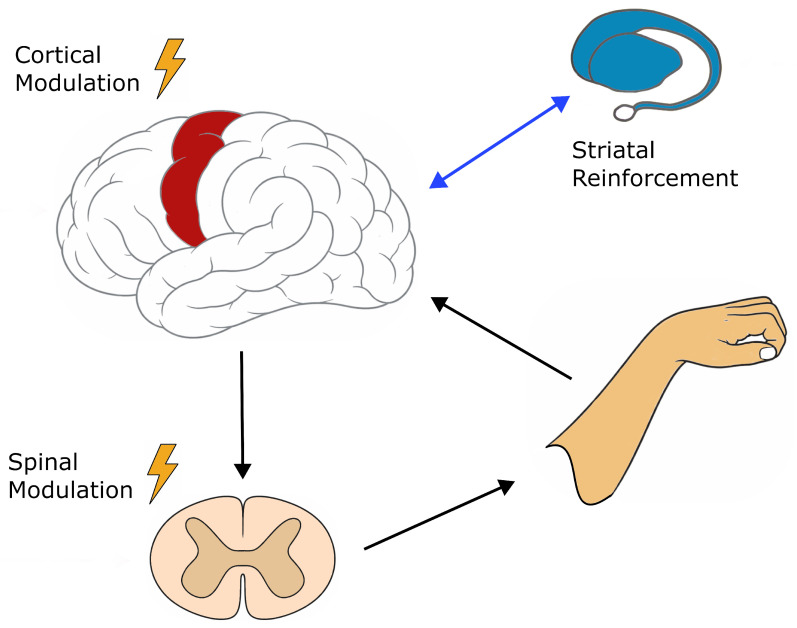
Multi-level motor hierarchy and sites of neuromodulatory intervention: Descending motor commands originate in primary motor cortex (M1, in red) and propagate through corticospinal pathways to spinal circuits, ultimately generating muscle activation. Sensory afferents convey peripheral feedback back to cortex, forming a closed sensorimotor loop that supports ongoing error correction and adaptation. In parallel, cortico-striatal–thalamo-cortical circuits provide reinforcement-dependent gating of cortical activity, stabilizing repeated patterns into consolidated motor behavior through dopamine-mediated reward signaling. Neuromodulatory interventions may act at multiple levels within this hierarchy: cortical stimulation aligns activity with endogenous oscillatory phase and state-dependent excitability (timing-dependent neuromodulation); striatal reinforcement reflects dopaminergic modulation of learning within basal ganglia circuits; and spinal stimulation increases segmental network responsiveness to descending input (segmental excitability modulation). Durable recovery is proposed to emerge when communication across these levels is temporally aligned and behaviorally contingent.

Similar effects are reported in humans. Bunday et al. [20] showed that precisely timed TMS volleys paired with peripheral input potentiate cortico-motoneuronal transmission, while Moritz et al. [108] reported parallel increases in cortical coherence and segmental excitability following dual-site stimulation. Such protocols enforce temporally aligned activation across cortical and spinal circuits, consistent with spike-timing–dependent plasticity principles. Repeated sessions may further entrain μ- and β-band oscillations and enhance cortico-muscular coherence, potentially facilitating reconnection of previously weakly coupled pathways [49,115]. Functionally, coordinated modulation at both levels may improve the reliability of descending motor output. Figure 1 summarizes this multi-level framework. By synchronizing excitability across the cortical–spinal axis, such approaches may stabilize communication within the injured motor hierarchy and create conditions favorable for durable learning.

## 7. Design Principles for Translational Neuromodulation

Neuromodulation appears most effective when applied within the same learning constraints that govern motor adaptation, rather than relying on stimulus intensity or waveform alone. The evidence reviewed above highlights four interacting constraints that influence whether stimulation promotes durable reorganization: temporal alignment with endogenous excitability states, behavioral contingency to provide reinforcement, engagement of the primary bottleneck within the motor hierarchy, and embedding within the temporal structure of learning.

First, stimulation must be temporally aligned with endogenous excitability states to engage Hebbian mechanisms. Plasticity is expressed within constrained windows defined by oscillatory phase, inhibitory tone, and recent activity history. Interventions delivered outside these receptive states may shift excitability transiently yet fail to induce lasting modification. Aligning stimulation with periods of heightened responsiveness increases the likelihood that induced activity coincides with intrinsic depolarization and supports stabilization of synaptic change.

Second, behavioral contingency determines whether facilitation consolidates into learning. Neuromodulation is most effective when stimulation is causally linked to volitional intent or task-relevant outcomes, allowing neural activity to predict sensory or motor consequences. This contingency converts stimulation into a reinforcement signal that stabilizes task-relevant patterns through feedback- and reward-dependent plasticity. Without such linkage, stimulation remains assistive rather than instructive, producing short-lived effects with limited generalization.

Third, intervention should target the dominant bottleneck within the motor hierarchy rather than be applied indiscriminately. Depending on lesion characteristics and residual circuitry, recovery may be constrained by impaired cortical initiation, weakened descending transmission, or disrupted cortical–spinal integration. Directing stimulation to the level that limits information flow—cortex, spine, or both—may amplify residual function and restore coordinated sensorimotor loops. In this framework, anatomical location is secondary to alignment with the principal constraint on recovery.

Finally, effective neuromodulation must respect the temporal structure of learning. Stimulation should be embedded within spaced, practice-driven training to influence consolidation rather than transient performance. Interventions synchronized with active learning and distributed across sessions are more likely to promote stabilization, whereas massed or poorly timed stimulation may saturate plastic mechanisms without durable benefit. Under these conditions, neuromodulation functions not as a generic excitability tool, but as a means of reinforcing learning within the injured motor hierarchy.

## 8. Discussion: Toward Mechanistically Informed Neurorehabilitation

Across the evidence reviewed, a central mechanistic theme becomes apparent: injury does not abolish plasticity, but shifts the conditions under which it can be expressed. Neuromodulation may therefore act not by creating new learning capacity, but by restoring access to mechanisms that remain functionally constrained. The findings summarized above identify measurable physiological signatures—such as μ- and β-band desynchronization, cortico-muscular coherence, and MEPs—that permit plasticity to be monitored in vivo. Incorporating these markers into control algorithms allows stimulation to be delivered preferentially during permissive neural states. In this framework, the design principles outlined earlier—temporal alignment, behavioral contingency, bottleneck targeting, and learning-consistent timing—can be implemented in real time. Closed-loop systems thus represent not a separate learning rule, but a practical strategy for maintaining stimulation within the viable range in which adaptive change can be expressed.

Translational design must account for inter-individual variability. Baseline excitability, inhibitory tone, and neuromodulator availability differ widely across patients and even across days within the same individual. Future systems will therefore require continual recalibration, adjusting phase, polarity, and dose to maintain stimulation within a safe and effective range. Real-time monitoring of cortical and spinal activity can help prevent excessive excitation while preserving engagement of residual circuits. In this context, metaplasticity may be viewed not only as a biological constraint but as a dynamic parameter that can be modulated to maintain responsiveness without inducing saturation.

Although the mechanistic rationale is robust, translation into consistent clinical benefit remains limited by heterogeneity and mixed outcomes at scale. Recent meta-analyses of BCI-based stroke rehabilitation report overall improvements in upper-limb motor function, while noting moderate methodological quality and substantial variability across trials, protocols, and patient cohorts, which constrains generalizability [116]. Similar syntheses of randomized controlled trials show significant average effects alongside meaningful between-study heterogeneity [117,118]. These patterns point to likely non-responder subgroups whose outcomes may depend on lesion characteristics, baseline neurophysiology, stroke chronicity, and task–feedback coupling—factors extending beyond CST integrity alone. Addressing this variability will likely require biomarker-informed stratification and adequately powered multicenter trials aligned with residual plastic potential.

Mechanistic precision must also be matched by ethical and practical scalability. Closed-loop neuromodulation systems introduce governance and safety challenges because stimulation parameters are dynamically adjusted rather than fixed by clinician-defined settings. Risks include unintended over-excitation, maladaptive plasticity, reduced user agency, and limited transparency in how algorithms translate neural signals into stimulation decisions. A recent scoping review of human closed-loop neurotechnology identified substantial variability in safety monitoring, stopping criteria, and disclosure of adaptive control logic across studies [119]. As these systems transition beyond specialized research settings, clear standards for algorithmic transparency, continuous physiological monitoring, and defined responsibility structures will be necessary to preserve autonomy and mitigate harm. Broader implementation will also depend on scalable hardware and regulatory alignment to ensure safe and equitable deployment.

In sum, advances in neurorehabilitation will likely depend less on stronger stimulation or increasingly complex devices and more on aligning intervention with the dynamic interaction between brain, body, and technology. Plasticity mechanisms often remain preserved after injury, but their expression is constrained by state, timing, and network integration. Designing interventions that account for these constraints may allow neuromodulation to function not merely as an adjunctive excitability tool, but as a mechanistically grounded component of learning and recovery.

## Data Availability

No new data were created or analyzed in this study. Data sharing is not applicable to this article.

## Glossary and Abbreviations

Plasticity window	A transient physiological state in which neural circuits exhibit enhanced susceptibility to activity-dependent synaptic modification
Hebbian plasticity	Synaptic learning mechanisms whereby coincident pre- and postsynaptic activity strengthens functional connectivity
Spike-timing-dependent plasticity (STDP)	Temporally precise form of Hebbian plasticity in which synaptic change depends on relative timing of pre- and postsynaptic firing
Long-term potentiation/depression (LTP/LTD)	Persistent increases/decreases in synaptic strength induced by specific patterns or timing of neural activity
Bienenstock–Cooper–Munro (BCM) model	A theoretical framework proposing a sliding modification threshold to balance synaptic stability and learning
Modification threshold ($\theta_{m}$)	A dynamic threshold that determines whether synaptic activity induces potentiation or depression, and shifts according to previous levels of neural activity
Homeostatic metaplasticity	Regulatory processes by which prior neural activity modulates the future capacity for synaptic plasticity
Closed-loop stimulation	Neuromodulation delivered in real time based on ongoing neural or behavioral signals
